# Burst that Bubble. Gastric Perforation from an Ingested Intragastric Balloon: A Case Report

**DOI:** 10.5811/cpcem.1668

**Published:** 2024-09-29

**Authors:** Miriam Martinez, Khristopher Faiss, Neha Sehgal

**Affiliations:** Texas Tech University Health Sciences Center at El Paso, Department of Emergency Medicine, El Paso, Texas

**Keywords:** intragastric balloon, gastric perforation, weight loss, case report

## Abstract

**Introduction:**

More than 40% of Americans are considered obese, resulting in annual healthcare costs estimated at $173 billion.^1^,^2^ Various interventions exist to address obesity including lifestyle modification, medications, and several surgical options. A novel ingestible intragastric balloon that self-deflates and is excreted approximately four months post-ingestion is being used in other countries such as Australia, Mexico, and several European countries. Currently, however, there are no US Food and Drug Administration-approved, commercially available options like this in the United States.

**Case Report:**

We present a case of a 31-year-old, obese male who presented to the emergency department for abdominal pain approximately 10 weeks after the ingestion of an inflatable balloon for weight loss treatment in Mexico. He was found to have a gastric perforation and required an emergent exploratory laparotomy.

**Conclusion:**

While ingestible, weight-loss balloons are not yet commercially available in the United States, emergency physicians may still encounter complications of such devices.

## INTRODUCTION

Obesity is an epidemic that is at the core of several common chronic health conditions including heart disease, type 2 diabetes, and hypertension. In 2022, the prevalence of obesity among adults in the United States was 41.9%. Black adults had the highest rate at 49.9%, followed by Hispanic adults at 45.6%.^1^ Nonsurgical and non-endoscopic ingestible, intragastric balloons (IGB) are being used in many countries including Mexico, Australia, and across Europe to assist patients with weight loss.

There are currently no US Food and Drug Administration-approved commercial options in the US. The primary benefits of ingestible IGBs are that they do not require anesthesia, sedation, or endoscopy for placement and subsequent retrieval. The balloon is ingested with a catheter attached; it is filled and then excreted in the stool approximately four months post-ingestion.^2^–^4^ For patients, this may be an attractive option as the device removes the risks associated with sedation, anesthesia, and endoscopy.^5^–^8^

## CASE REPORT

A 31-year-old obese, Hispanic male presented to the emergency department with progressively worsening left upper quadrant abdominal pain for five days. The pain was described as cramping, intermittent, and exacerbated by bending forward. He denied past medical history.

Several days prior to presentation the patient had been evaluated by his primary care physician. He was diagnosed with musculoskeletal pain and prescribed cyclobenzaprine. Since the evaluation by the primary care physician, the pain had increased in frequency and intensity. The patient denied any nausea, vomiting, fevers, diarrhea, or hematochezia. He did, however, report intentional weight loss of 15 kilograms. Ten weeks prior, the patient had ingested an IGB (Allurion, formerly known as Elipse), for weight-loss purposes under the care of a physician in Juárez, Mexico.

On initial evaluation, vital signs were normal except for sinus tachycardia at 105 beats per minute. He was in mild distress with moderate tenderness to palpation in the left upper quadrant. There was no guarding, rebound, or rigidity. Laboratory evaluation showed a white blood cell count of 21.7 × 10^3^ cells per cubic millimeter (mm^3^) (reference range 4.5 – 11 × 10^3^ cells/mm^3^) with a neutrophil predominance of 18.9 × 10^3^ cells/mm^3^ (2 – 7.8 × 10^3^ cells/mm^3^) and an elevated blood urea nitrogen at 25 milligrams (mg)/deciliter (dL) (9–20 mg/dL). The patient’s electrolytes, liver function tests, and serum creatinine were within normal limits. A computed tomography (CT) of the abdomen and pelvis with intravenous contrast was ordered to assess for bowel obstruction, perforation, and balloon integrity. The CT indicated intraluminal gastric balloon with anterior gastric wall perforation without evidence of intestinal pathology (Image).

General surgery was consulted and obtained a CT abdomen and pelvis with oral contrast, which directly showed extravasation into the peritoneal cavity. The patient was emergently taken to the operating room for an exploratory laparotomy, abdominal washout, removal of the IGB, and gastric perforation repair. He was started on piperacillin/tazobactam and fluconazole for purulent peritonitis. After an overnight stay in the surgical intensive care unit, he was transferred to the surgical floor where he remained for the following 10 days. On day 5, a follow-up upper gastrointestinal series with gastrografin was obtained, and no contrast extravasation was noted. The patient’s diet was advanced, and he was discharged on postoperative day 10 with amoxicillin-clavulanate for 10 days. Forty-two days after discharge the patient was noted to be recovering well on a follow up visit.

## DISCUSSION

Our case demonstrates a potentially lethal complication of this novel ingestible IGB. The device is different from other tools for weight loss in that it does not require surgery, endoscopy, or anesthesia for placement and removal. It also is more convenient in that placement can be done in a 20-minute visit.^5^ The balloon takes up volume within the stomach, promoting early satiety and weight loss.^3^–^8^

**CPC-EM Capsule**

What do we already know about this clinical entity?

*Various interventions exist to address obesity. An ingestible, intragastric balloon that self-deflates and is excreted four months post-ingestion is not yet available in the United States (US).*

What makes this presentation of disease reportable?

*An obese male presented to our emergency department with a gastric perforation 10 weeks after ingestion of a weight-loss balloon in Mexico.*

What is the major learning point?

*Complications from ingestible, intragastric balloons including perforations are more likely to occur on the US-Mexico border or in places with a large medical tourism population.*

How might this improve emergency medicine practice?

*Emergency physicians should consider gastric perforation in patients with intragastric balloons. Early specialist involvement is key to managing such patients.*

The IGB balloon is made from biodegradable materials and folded into a vegan capsule. While still connected to a thin filling catheter the capsule with the IGB inside is swallowed. Positioning in the stomach is confirmed with a radiograph, and the balloon is filled with 550 milliliters of distilled water, potassium sorbate, and citric acid as preservatives with a reconfirmation of positioning following the filling of the balloon. Once the balloon is filled, the catheter is pulled out and the fill valve made from a thin film is sealed shut. The IGB has a self-release valve that is closed by a filament that is only in contact with the inside of the balloon. This filament weakens gradually and breaks at approximately four months. Breaking of the filament causes the contents of the balloon to empty into the stomach. Once the balloon is emptied it is excreted in a bowel movement.^4^–^8^

Literature on the device is limited as the device is mostly being used in the Middle East and Europe. Six unique studies comprised of 2,013 patients were evaluated in a systematic review and meta-analysis on the efficacy and safety of the device. This study found that all the balloons had successful placement except for one that was retained in the lower portion of the esophagus. While there was a small number of serious complications no mortalities were reported.^8^ Among the 2,013 cases there was only one with a gastric perforation that required surgery.^5^–^8^ Other complications, which were also rare, included small bowel obstructions in three patients, spontaneous balloon hyperinflation in four patients, and gastric outlet obstruction, pancreatitis, and esophagitis, each in one patient.^4^,^8^ While this balloon has an overall favorable safety profile it is important to note that there is still the possibility of serious complications. To our knowledge this case presents only the second instance of a gastric perforation from an IGB.

## CONCLUSION

Gastric perforation is a rare but serious complication associated with a high mortality rate in patients with intragastric balloon placement. Complications from such devices are more likely to occur on the US-Mexico border or in communities with a large medical tourism population, as the procedure is not available in the United States. It is prudent for emergency physicians to consider gastric perforation in patients with intragastric balloons. Early specialist involvement and prompt resuscitation are key to successfully managing such patients.

## Figures and Tables

**Image:**
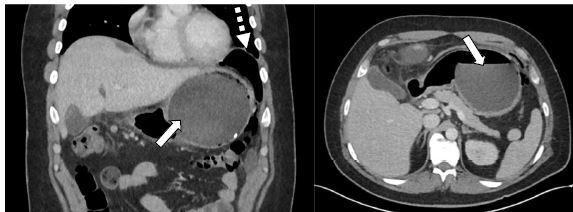
Computed tomography of the abdomen and pelvis, coronal view (left) and axial view (right), with intravenous contrast demonstrating intraluminal gastric balloon (solid arrow) with perforation and free air in the left upper quadrant (dashed arrow).
